# Shared Decision-Making and Role Preference Among Patients With Schizophrenia in Malaysia: A Cross-Sectional Study

**DOI:** 10.3389/fpsyt.2021.680800

**Published:** 2021-07-26

**Authors:** Mohamad Ayob Ismail, Marhani Midin

**Affiliations:** Psychiatry Department, Universiti Kebangsaan Malaysia Medical Center (UKMMC), Kuala Lumpur, Malaysia

**Keywords:** shared decision making, role preference, schizophrenia, associated factors, Malaysia

## Abstract

**Introduction:** Shared decision-making (SDM) is recognized as a promising strategy for improving collaboration between clinicians and their patients in achieving recovery. In Malaysia, SDM among people with schizophrenia is still lacking both in practice and in research. This study aimed to determine the level of SDM and role preference and their associated factors among patients with schizophrenia in Malaysia.

**Methods:** A cross-sectional study was conducted on 86 outpatient attendees with schizophrenia at a teaching hospital in Kuala Lumpur, Malaysia. The nine-item Shared Decision Making Questionnaire and Control Preference Scale were used to assess perceived SDM experience and role preference, respectively. Linear and logistic regression models were used to analyze the factors associated with SDM and role preference, respectively. Factors with a p <0.25 from the simple regression analyses were controlled as the covariates in the multiple regression analyses.

**Results:** The study respondents were predominantly female, single, and unemployed, with a mean age of 44 years. Only 35% of the participants reported having high SDM experiences, even though the majority (56%) preferred autonomous role preference. Among the participants who preferred autonomous roles, only 40% experienced high SDM. High SDM was found to be significantly associated with being younger (B = −0.33, 95% CI = −0.67 to −0.003) and being non-clozapine users (B = 19.90, 95% CI = 9.39–30.41), while autonomous role preference was significantly associated with a lower level of insight [adjusted odds ratio (AOR) = 0.84, 95% CI = 0.72–0.99] and being on oral antipsychotic drugs only (AOR = 2.94, 95% CI = 1.10–7.82).

**Conclusion:** The practice of SDM is still lacking in the treatment of patients with schizophrenia in Malaysia, even though many of them preferred to be involved in the decision-making pertaining to their treatment. This study indicates the need for clinicians to improve their patients' involvement in the treatment process. More research is needed on how SDM can be implemented in patients with schizophrenia, especially in Asian population settings.

## Introduction

Shared decision-making (SDM) is recognized as a promising strategy for improving collaboration between clinicians and patients in achieving recovery. SDM is a process in which clinicians and patients work together to select tests, treatments, and management or support packages based on clinical evidence and the patient's informed preferences. It involves the provision of evidence-based information about options, outcomes, and uncertainties, together with decision support counseling and a system for recording and implementing patients' informed preferences (1). It was developed in the mental health field in response to the reality that psychiatric medications come in a package with varying degrees of benefits and risks. Therefore, there is a need for a process that ensures concordance between clinicians and their patients (2). SDM has been shown to improve functional outcomes (3) and quality of life (4) and enhance satisfaction and adherence with medication among all patients, in general patient population (5). Similar outcomes were observed in patients with mental illnesses, with the added benefits of reduced anxiety and depression following SDM interventions (6). Furthermore, SDM has been shown to improve personal recovery among patients, and its application has been suggested in the broader context of decision-making related to rehabilitation (7). One recent cost–benefit analysis study on pharmaceutical care among patients with schizophrenia revealed a net benefit of more than USD 2,000 within 3 months when SDM intervention was practiced (8).

SDM largely reflects the values in medical practices in western countries in Europe and North America (9). In Malaysia, research on SDM was initiated in 2010–2011 (10) and considered a pioneering work in Asia (11). Existing local studies showed low levels of patient and public involvement in SDM. A study showed that doctors were aware of informed consent, but few practiced SDM (12). Another study revealed a lower rate of preference for SDM among rural as compared to urban population (28 and 51%, respectively) (13). There has been an increasing recognition and effort from the academia and the Health Ministry to follow the first steps in SDM with patient involvement (14). Additionally, SDM has become more widely discussed in recent years in Malaysia and other non-Western countries including China, Taiwan, and Iran (14). In China, it was reported that information about SDM is still limited with very sparse evidence—qualitative or quantitative—about the feasibility, cultural and structural fit, processes, and outcomes of SDM (11). A study indicated that doctor–patient relationships are poor, consultations are brief, and levels of trust are low (14). It was concluded that implementing SDM that involves a shift in doctor–patient power balance may be challenging in Asian countries like China and Malaysia (11, 14).

SDM in mental health has started to gain mileage in Malaysia only in very recent years. A locally developed intervention to promote SDM was created in 2017 involving the use of antidepressants among patients with major depressive disorder (MDD) (15). Research to determine its effectiveness is currently undergoing. Particularly among patients with schizophrenia, SDM approach is still lacking both in practice and in research in Malaysia. Available studies in other countries generally revealed inconsistent and inadequate SDM involvement of patients with schizophrenia in their treatment and care (16, 17). One study was a randomized controlled trial in Japan (18), which was prematurely terminated due to slow enrollment. A recent qualitative study in China revealed main themes of patients having a positive attitude and self-motivation in decision-making but feeling excluded from the process (19). SDM experience is generally lower among patients with schizophrenia than those with milder conditions. A study done in Spain reported lower rates of SDM experience among patients with schizophrenia as compared to others with bipolar disorder, depressive disorder, and anxiety disorder (10, 15, 17, and 18%, respectively) (20). Among all patients with different psychiatric diagnoses, a study reported 60% SDM experience at some point in their care (21).

An important concept related to SDM is role preference, as not all patients may desire or are prepared to participate in the treatment decision-making process with their physicians (22). Some patients want active or shared responsibility, while others may be passive decision makers who prefer their providers to make treatment choices and decisions on their behalf. There is a wide variation of reported role preference among patients with psychiatric illness. A study in Spain revealed that only 36% of patients with bipolar disorder and schizophrenia preferred autonomous roles (23). Other studies reported higher levels of role preference among patients with mental illness (24). For example, a review on published surveys showed that a majority of patients wanted a shared responsibility on their healthcare decisions with their doctors (25). A very recent study revealed 82% of mental health service users preferred autonomous roles (26). SDM experiences and role preference may be associated with multiple factors, such as demographic variables, clinical characteristics, and types of clinical decisions (25, 27). Among the sociodemographic factors, being younger (20) and having a higher educational level and economic status (28) were known to be associated with autonomous role preference in decision-making. The level of insight among patients with schizophrenia was shown in a study to have the strongest link to a poor decision-making capacity among all clinical characteristics (29). Patients may prefer active roles in types of decisions relating to behavioral changes, less serious illnesses, and lifelong decisions while preferring passive roles in decisions concerning severe exacerbations of a condition (27).

While there is ample evidence indicating its benefits, SDM implementation for patients with serious mental illness has been relatively less successful than for other groups of patients (30). Individuals with schizophrenia, among all the patients with mental illnesses, experience the lowest SDM (20). This could be due to many possible barriers in implementing SDM in this group of patients. Clinicians may have the assumption that individuals with schizophrenia lack the capacity for decision-making in their treatment (18). Schizophrenia, by nature, is a chronic and disabling illness, with the majority of patients experiencing multiple relapses during the course of the illness (31). Common symptoms like delusions, apathy, and social withdrawal, which can affect relationships and desire to take part in decision-making, may present as significant therapeutic barriers to SDM (32). To the best of our knowledge, there was no published study on SDM and role preference among patients with schizophrenia in Malaysia at the moment this study was conducted. The findings from this study would add to the scientific data in countries that are less represented in the SDM research and practice to facilitate its implementation, measurements, and interventions. In this study, we aimed to determine the level of SDM and role preference and their associated factors among patients with schizophrenia in Malaysia.

## Materials and Methods

### Study Setting and Design

This study was conducted among patients with schizophrenia at the Universiti Kebangsaan Malaysia Medical Center (UKMMC). The UKMMC is an academic medical center that was created by the merger of the Faculty of Medicine and the teaching hospital of Universiti Kebangsaan Malaysia (UKM) and is located in Kuala Lumpur, Malaysia. This cross-sectional study was conducted from July 2020 to January 2021 at the outpatient psychiatric clinic of the UKMMC during patients' follow-up visits.

### Population and Sample

The inclusion criteria were individuals attending the psychiatry clinic during the study period who (1) were diagnosed as having schizophrenia by an experienced psychiatrist based on the Diagnostic and Statistical Manual of Mental Disorders, Fifth Edition (DSM-5); (2) were aged 18 years and above; (3) had a sufficient command of both English and Bahasa Malaysia (the national language); (4) were clinically stable, as judged by their treating psychiatrist, i.e., they were treated as outpatients, had no modified treatment regimen, and had had no essential change in symptomatology for at least the previous 6 months (33). The exclusion criteria were those who (1) were exhibiting aggressive behavior, (2) had concomitant intellectual disability, (3) had severe cognitive impairment, (4) refused informed consent, (5) were not clinically stable (33).

### Study Instruments

Four instruments were used in this study.

#### Sociodemographic and Clinical Characteristics Questionnaire

This is a researcher-generated questionnaire that captures sociodemographic information: age, gender, ethnicity, religion, marital status, level of education, and employment status. The clinical characteristic variables were as follows: the age of onset, the duration of untreated psychosis (DUP) as within or more than a year (34), the duration of illness, the number of psychiatric hospitalizations, and antipsychotic treatment. Antipsychotic treatments were assessed for the route of administration [only oral or with long-acting injectable (LAI) antipsychotics] and types of antipsychotics. In this study setting, second-generation antipsychotics (SGAs) are the most prescribed type of antipsychotics (35). Clozapine is used in this center for treatment-resistant schizophrenia (TRS).

#### The Nine-Item Shared Decision Making Questionnaire

Measurements for SDM can be categorized by decision antecedents (role preference), the decision process (observed or perceived behavior of the clinician), or decision outcomes (decisional conflict or satisfaction) (11). Few scales are available that assess SDM from both the patient's and the physician's points of view. This includes the nine-item Shared Decision Making Questionnaire (SDM-Q-9), which was published in 2010 (32) and is commonly used to assess interventions aiming to improve SDM. The SDM-Q-9 has good psychometric testing and acceptance and is relatively easy to administer with only nine items (34). Internal consistency yielded a Cronbach's alpha of 0.938 (36). It is a patient-reported measure that focuses on the decisional process by rating physicians' and patients' behavior in medical encounters. It was developed as a revision of the original Shared Decision-Making Questionnaire in 2006 (32). Response options were provided in the form of a 6-point Likert scale ranging from “completely disagree” (0) to “completely agree” (5) for each item. Summing up all of the nine items leads to a raw total score between 0 and 45. Multiplying the raw score by 20/9 provides a score forced (transformed) to range from 0 to 100, where 0 indicates the lowest possible level of SDM and 100 indicates the highest extent of SDM. Various studies have used different cutoffs, as there were no predefined cutoffs. One study transformed into three categories using tertiles of the theoretical score range: (1) low SDM, with SDM-Q-9 sum scores up to 33; (2) intermediate SDM, with SDM-Q-9 sum scores between 34 and 66; and (3) high SDM, with SDM-Q-9 sum scores of at least 67 (37). SDM dichotomous variables were computed as total scores of percentile 25 or lower representing a low perception of SDM and percentile 75 and above as having a high perception of SDM in another study (20). Due to pragmatic considerations (the variation of cutoffs), percentile 75 and above was analyzed as high SDM in this study. Meanwhile, total scores 0–100 were used as a continuous variable for the inferential analyses. SDM-Q-9 is available and validated in a range of different languages, including the English and Malay versions. It is accessible to be downloaded from www.sdmq9.org as public domain software. Written permission was granted to use SDM-Q-9 in this research. The SDM-Q-9-Psy (Hebrew) scale for evaluating SDM from the perspective of psychiatric inpatients was also developed with good reliability and validity (38, 39).

#### Control Preference Scale

This scale determines the degree of control a patient wants to assume when decisions are being made about medical treatment (40). It consists of one question: “How do you prefer to make a decision during consultation?” and has five options in terms of answers to choose from: option 1 = “I prefer to make the final treatment selection about which treatment I receive”; option 2 = “I prefer to make the final selection of my treatment after seriously considering my doctor's opinion”; option 3 = “I prefer that my doctor and I share responsibility for deciding which treatment is best for me”; option 4 = “I prefer that my doctor makes the final decision about which treatment will be used but after seriously considering my opinion”; option 5 = “I prefer to leave all the decisions regarding my treatment to my doctor.” Autonomous and passive role preferences were determined by regrouping the chosen options, i.e., option 1 or 2 or 3 became an autonomous roles preference, while option 4 or 5 became a passive role preference. A Cronbach's alpha of 0.72 was attained, pointing out a moderate internal consistency level (41). Permission to use this scale was granted for this study. The validated Malay version (13) of the scale was used with permission.

#### Schedule for the Assessment of Insight

The Schedule for the Assessment of Insight (SAI) is an interviewer-rated, three-item rating scale used to evaluate insight into psychotic illness (42). The SAI assesses insight in three domains: Awareness that one has a mental illness [0–6], Ability to relabel psychotic phenomena as symptoms of mental illness (0–4), and Awareness of the need for treatment (0–4). Respondents are scored on a 0–2 scale (0 = never, 2 = often). The total score is 14, with higher scores indicating a higher level of insight. The SAI has the advantage of brevity and ease of administration. This questionnaire has been widely used among patients with psychoses (43). A comparative study of various insight scales demonstrated a high correlation between the SAI and the other insight measurement scales (44). This suggests that the SAI has good concurrent validity. Written permission to use this scale was granted by the original author.

### Study Procedure and Data Collection

Eligible participants were identified at the clinic triage counter from the daily registration book and patients' medical records. Patients attending the psychiatry clinic of the UKMMC during the data collection period were approached in the waiting area while they were waiting to be seen. A total of 112 respondents were approached. A total of 26 participants were excluded from the study for several reasons: 11 had difficulty comprehending English or Malay, eight were rushing to leave the clinic, five refused to participate without giving any reason, and two had prominent psychotic symptoms with persistent irrelevant speech. The response rate was 76.8%, producing a final sample of 86.

Each participant received a full written explanation of the study, after which they signed an informed consent form. Each patient was given all four questionnaires. These were self-administered with the assistance of the researcher or caregiver except for the SAI questionnaire, as it was interviewer-rated. To rate the SDM-Q-9, participants were instructed to think about their last consultation and to use this event as a reference point for the rating. Patients received no financial compensation for their participation.

### Statistical Analysis

Data were entered and analyzed using IBM SPSS Statistics 26. For the categorical variable, descriptive data were presented by absolute number and percentage. For the continuous variable, descriptive data were expressed as mean ± standard deviation (SD) or median ± interquartile range (IQR) depending on the normality of the data. The normality of the distribution of the continuous variables was evaluated using a histogram and the Shapiro–Wilk normality test. Simple linear regression (SLR) analysis was done to determine the important independent variables for the SDM total scores as a continuous dependent variable. Meanwhile, simple logistic regression (SLogR) analysis was done for the Control Preference Scale (CPS) level as a dichotomous dependent variable. The variables with a p <0.25, or any clinically important factors, were selected for multiple linear and multiple logistic regression (backward method). Those with a p <0.05 were considered statistically significant. Multicollinearity, interaction, and model fit analyses were also performed on the model.

### Ethical Considerations

This study was approved by the UKM research ethics committee (JEP-2019-530). Informed consent was obtained from each patient before the study was conducted and after an explanation of the purpose of the study and assurance of the confidentiality of individual data collected. All clinical data were kept in a secure, password-protected electronic database system.

## Results

### Descriptive Analysis

A total of 86 patients participated in the study. The mean age for the respondents was 44.86 (SD = 13.86) years. The majority of them were female (60.5%). Regarding ethnicity, Malay was the highest number of participants [(38), 44.2%], slightly higher than Chinese [(35), 40.7%]. The majority were single [(45), 61.6%], which included those never married, divorced, and widowed. Most [(46), 53.5%] had up to secondary education. At least 29 (33.7%) of them had at least tertiary education of certificate/diploma and above. The majority were unemployed [(47), 70.9%]. Among 25 participants who were employed included an intensive care unit (ICU) nurse, lecturer, teacher, and real estate negotiator. Details of the sociodemographic aspects of the study sample are provided in Table 1.

**Table 1:** Sociodemographic characteristics of respondents (*N* = 86).

**Variables**	**Mean (SD)**	***N***	**%**
			
Age	44.86 (13.86)		
Gender			
Male		34	39.5
Female		52	60.5
Race			
Malay		38	44.2
Chinese		35	40.7
Indian		13	15.1
Religion			
Islam		38	44.2
Buddhist		25	29.1
Christian		13	15.1
Hindu		10	11.6
Marital status			
Single		53	61.6
In marriage		33	38.4
Occupation			
Employed		25	29.1
Unemployed		61	70.9
Educational level			
No/Primary education		11	12.8
Secondary education		46	53.5
Certificate/Diploma		19	22.1
Undergraduate/Postgraduate		10	11.6

*SD, standard deviation*.

The clinical characteristics of the respondents, SDM scores, role preference level, and SAI are summarized in Table 2. A total of seven respondents could not recall their age of onset of symptoms. Thus, only 79 respondents completed the questions related to the age of onset, duration of untreated psychosis, and the duration of the illness. The median age of onset was 25 years. Only 30.8% of respondents had DUP within a year. The median duration of illness was 18 years. A total of 33.7% of respondents had no history of psychiatric hospitalization, while 11.7% had been hospitalized more than five times. In addition, 65.1% of respondents had only oral antipsychotics as the route of administration, while 25.6% had clozapine as one of the antipsychotic treatments. The SAI median scores were 10 out of 14 as the overall total scores for the level of insight.

**Table 2:** Clinical characteristics, SDM scores, CPS level, and SAI scores.

**Variables**	**Mean (SD)**	**Median (IQR)**	***N***	**%**
Age of onset		25.00 (15)		
Before 18 years old			16	20.3
18–30 years old			42	53.2
31–40 years old			9	11.4
After 40 years old			12	15.2
DUP				
Within a year			30	38.0
More than a year			49	62.0
Duration of illness (years)		18.00 (15)		
Number of Psychiatric Hospitalization				
Never			29	33.7
1–5 times			47	54.7
6–10 times			6	7.0
More than 10 times			4	4.7
Antipsychotics treatments				
Route of administration				
Oral only			56	65.1
With LAI			30	34.9
Type of antipsychotic				
No clozapine			64	74.4
With clozapine			22	25.6
SDM total scores	62.09 (22.76)			
High SDM (75 and more)			30	34.9
>75			56	65.1
CPS level				
Autonomous			48	55.8
Passive			38	44.2
SAI total scores		10.00 (5)		

*CPS, Control Preference Scale; DUP, duration of untreated psychosis; IQR, interquartile range; LAI, long-acting injectable; SAI, Schedule for the Assessment of Insight; SDM, shared decision-making*.

The mean SDM total score was 62.09 (SD = 22.76). Only 34.9% of respondents scored high SDM. A total of 65.1% of respondents scored below 75 for SDM. A total of 55.8% of respondents had autonomous role preference, while 44.2% preferred to be passive. Among these 48 respondents who preferred autonomous role preference, 29 (60.4%) had not scored high SDM. Meanwhile, only 19 respondents matched their autonomous role preferences with a high SDM total score, which represented only 22.1% of the total of 86 participants.

### Inferential Analysis

#### Simple Linear and Multiple Linear Regression Analyses to Determine the Factors Associated With the Shared Decision-Making Total Score

Simple linear regression analyses were used to determine the factors associated with the SDM total scores. Significant associations observed were between the SDM with the SAI total scores (*p* = 0.029) and no clozapine usage (*p* = 0.001).

Multiple linear regression analysis was conducted, with variables showing *p* <0.25 or any clinically important factor from the simple linear regression analysis. The independent variables selected were age, religion, education level, duration of illness, number of psychiatric hospitalizations, antipsychotic treatments (LAIs or oral only and with or without clozapine), the SAI total scores, and CPS level. These covariates were controlled in the multiple linear regression.

During Step 1, all selected independent variables were entered and explained 24.2% of the variation in the SDM total scores as the initial r-square. In Steps 2, 3, and 4, duration of illness, Hindu religion, and LAI antipsychotics were removed with no r-square changes. In Steps 5 and 6, psychiatric hospitalizations of 6–10 times and educational level were removed with both explained 24.1% of r-square. In Steps 7, 8, 9, 10, and 11, psychiatric hospitalizations of 1–5 times, SAI total scores, Buddhist religion, psychiatric hospitalizations of more than 10, and CPS level were removed, respectively. R-square changes were from 23.5, 23.0 22.3, 21.3 to 19.7% respectively. In Step 12, the Christian religion was removed and left with age and clozapine usage as the significant predictors with an overall r-square of 18.2%, which means that there are other factors relating to SDM total scores that have not been included in this study.

Two significant factors associated with SDM total scores were identified while other factors were being controlled. It was observed that age (B = −0.334, 95% CI = −0.666 to −0.003) was found to have a significant negative correlation, while “being a non-clozapine user” (B = 19.899, 95% CI = 9.392–30.406) was found to have a significant positive correlation with the SDM total scores. Table 3 shows the results of factors associated with SDM using simple linear regression and multiple linear regression.

**Table 3:** Factors associated with SDM using SLR and multiple linear regression.

**Variables**	**SLR**		**Multiple Linear Regression**		
	***b*^a^ (95% CI)**	***p*-value**	**Adj. b (95% CI)**	***t*-stat**	***p*-value**
Age	−0.281 (−0.640, 0.77)	0.122	−0.334 (−0.666, −0.003)	−2.010	**0.048**
Gender					
Male	Ref				
Female	1.301 (−8.739, 11.341)	0.797			
Race					
Malay	Ref				
Chinese	−5.709 (−15.756, 5.599)	0.347			
Indian	−1.881 (−16.525, 12.762)	0.799			
Religion					
Islam	Ref				
Buddhist	−8.533 (−20.215, 3.150)	0.150	−4.395 (−15.332, 6.543)	−0.801	0.426
Christian	1.881 (−12.695, 16.457)	0.798	7.653 (−5.323, 20.629)	1.175	0.244
Hindu	−1.335 (−17.458, 14.788)	0.870	0.670 (−16.955, 18.294)	0.076	0.940
Marital status					
In marriage	Ref				
Single	−0.210 (−10.308, 9.889)	0.967			
Occupation					
Employed	Ref				
Unemployed	4.706 (−6.061, 15.472)	0.387			
Educational level					
Up to secondary education	Ref				
College/university	8.750 (−1.463, 18.962)	0.092	1.396 (−10.368, 13.160)	0.237	0.814
Age onset	−0.110 (−0.523, 0.303)	0.596			
DUP					
Within a year	Ref				
More than a year	−5.346 (−15.728, 5.036)	0.308			
Duration of illness	−0.345 (−0.792, 0.103)	0.129	0.002 (−0.585, 0.589)	0.006	0.995
					
No. of psychiatric hospitalization					
Never	Ref				
1–5 times	−0.405 (−11.035, 10.225)	0.940	3.490 (−13.714, 6.336)	−0.694	0.490
6–10 times	−1.676 (−21.866, 18.513)	0.869	1.851 (−18.977, 22.679)	0.177	0.860
More than 10 times	−23.526 (−47.536, 0.485)	0.055	−11.396 (−34.60, 11.807)	−0.979	0.331
Antipsychotics treatments					
Route of administration					
With LAI	Ref				
Oral only	−6.795 (−16.993, 3.403)	0.189	−0.794 (−11.974, 10.385)	−0.142	0.888
Type of antipsychotic					
With clozapine	Ref				
No clozapine	18.422 (7.901, 28.944)	**0.001**	19.899 (9.392, 30.406)	3.772	**0.000**
SAI total scores	1.789 (0.184, 3.394)	**0.029**	0.630 (-1.149, 2.409)	0.706	0.483
CPS level					
Autonomous	Ref				
Passive	−5.740 (−15.550, 4.069)	0.248	−5.750 (−15.100, 3.600)	−1.225	0.224

*Bold values indicate Significant p < 0.05*,

a *crude regression coefficient. Multivariate linear regression (R^2^ = 0.182; the model reasonably fits well; model assumptions are met; there is no interaction between independent variable and no multicollinearity problem)*.

*CPS, Control Preference Scale; DUP, duration of untreated psychosis; LAI, long-acting injectable; SAI, Schedule for the Assessment of Insight; SDM, shared decision-making; SLR, simple linear regression*.

#### Simple Logistic and Multiple Logistic Regression Analyses to Determine the Factors Associated With the Autonomous Role Preference Level

Simple logistic regression analyses were used to determine the factors associated with autonomous role preference. No significant association was observed with autonomous role preference from simple logistic regression. However, five variables had a p <0.25.

Multiple logistic regression analysis was conducted with these five variables. The independent variables selected were age, number of psychiatric hospitalizations, antipsychotic treatments (LAI or oral only), the SAI, and SDM total scores. These covariates were controlled in the multiple logistic regression. The model fit the sample as a Hosmer and Lemeshow test showed a p = 0.634. During Step 1, all selected independent variables were entered and explained 17.6% of the variation in the CPS level as the initial Nagelkerke r-square. In Step 2, psychiatric hospitalization of 6–10 times was removed with no r-square changes. In Steps 3, 4, and 5, SDM total scores, age, and psychiatric hospitalizations of more than 10 were removed respectively. Nagelkerke r-square changes were from 16.9, 14.9, to 12.9% respectively. In Step 6, psychiatric hospitalization of 1–5 times was removed and left with SAI total scores and LAI usage as the significant predictors with an overall r-square of 11.3%, meaning there are other factors for role preference level that have not been included in this study.

Two significant factors associated with autonomous role preference were identified while other factors were being controlled. Every one increment of the SAI total scores decreases by 0.84 times the probability of having autonomous role preference [adjusted odds ratio (AOR) = 0.844, 95% CI = 0.719–0.989]. Those using the oral route only in the administration of antipsychotics had 2.94 times the probability of having autonomous role preference compared to those who had LAI antipsychotics (AOR = 2.939, CI = 1.104–7.823). Table 4 shows the results of factors associated with role preference using simple logistic regression and multiple logistic regression.

**Table 4:** Factors associated with role preference using SLogR and multiple logistic regression.

**Variables**	**SLogR**		**Multiple Logistic Regression**		
	**Crude OR (95% CI)**	***p*-value**	**Adj. OR (95% CI)**	**Wald**	***p*-value**
Age	1.022 (0.989, 1.055)	0.189	1.017 (0.983, 1.051)	0.945	0.331
Gender					
Male	1				
Female	0.669 (0.277, 1.61)	0.370			
Race					
Malay	1				
Chinese	1.440 (0.398, 5.211)	0.578			
Indian	1.200 (0.326, 4.414)	0.784			
Religion					
Islam	1				
Buddhist	2.100 (0.471, 9.364)	0.331			
Christian	1.833 (0.383, 8.778)	0.448			
Hindu	2.000 (0.352, 11.364)	0.434			
Marital status					
In marriage	1				
Single	1.087 (0.453, 2.606)	0.852			
Occupation					
Employed	1				
Unemployed	0.621 (0.238, 1.619)	0.330			
Educational level					
Up to secondary education	1				
College/university	1.188 (0.481, 2.935)	0.709			
Age onset	1.014 (0.978, 1.052)	0.437			
DUP					
Within a year	1				
More than a year	0.864 (0.346, 2.157)	0.755			
Duration of illness	1.014 (0.974, 1.055)	0.498			
No. of Psychiatric Hospitalization					
Never	1				
1–5 times	0.175 (0.016, 1.913)	0.153	0.192 (0.015, 2.496)	1.590	0.207
6–10 times	0.319 (0.031, 3.297)	0.338	0.396 (0.031, 5.021)	0.511	0.475
More than 10 times	0.167 (0.010, 2.821)	0.214	0.212 (0.011, 4.181)	1.041	0.308
Antipsychotics treatments					
Route of administration					
With LAI	1				
Oral only	2.179 (0.884, 5.373)	0.091	2.939 (1.104, 7.823)	4.658	**0.031**
Type of antipsychotic					
With clozapine	1				
No clozapine	0.836 (0.313, 2.231)	0.720			
SAI total scores	0.886 (0.764, 1.027)	0.107	0.844 (0.719, 0.989)	4.375	**0.036**
SDM total scores	0.989 (0.970, 1.008)	0.246	0.992 (0.971, 1.014)	0.476	0.490

*Bold values indicate Significant p < 0.05, 1, reference. Multiple Logistic Regression: Cox & Snell R Square 8.4%, Nagelkerke R Square 11.3%; the model reasonably fits well; model assumptions are met; there is no interaction between independent variable and no multicollinearity problem*.

*DUP, duration of untreated psychosis; LAI, long-acting injectable; OR, odds ratio; SAI, Schedule for the Assessment of Insight; SDM, shared decision-making; SLogR, simple logistic regression*.

## Discussion

This study aimed to determine the level of SDM and role preference and their associated factors among patients with schizophrenia. To our knowledge, this is the first study that examined SDM among patients with schizophrenia in Malaysia. Overall, this study yielded four main findings. First, 35% of the study participants experienced high SDM, and 56% preferred autonomous roles. Second, role preference did not correlate well with SDM experiences; the majority of participants who preferred autonomous roles perceived a lack of SDM. Third, being younger and a non-clozapine user were factors significantly associated with SDM experiences. Fourth, a lower level of insight and being on oral antipsychotics only were significantly associated with autonomous role preference.

### Level of Shared Decision-Making and Role Preference

#### Level of Shared Decision-Making

The majority (65%) of the study participants perceived a lack of SDM experiences, whereas only 35% experienced a good level of SDM with a mean score of 62. Studies of SDM among patients with schizophrenia remain lacking. The majority of the research on SDM in the mental health field has focused on mental illness in the population in general and has been done mainly in the United States and European countries (48). The only study to which we can compare our findings is a study done in Spain (20) that focused on patients with schizophrenia and used the same measurement tool and cutoff point. This study revealed an even lower percentage of participants experiencing a good level of SDM (10%), with a mean score of 39. Otherwise, the study on patients with all psychiatric conditions revealed a much higher percentage (60%) of participants experiencing a good level of SDM at some point in their care (21). A recent national survey in Hungary that studied the general adult population using the same measurement tool revealed a higher mean score of 67 (46).

Other studies on SDM among people with schizophrenia are qualitative in nature, which focused on an exploration of the elements of SDM. One qualitative observational study on psychiatric illness, with patients with schizophrenia as the majority of the participants, revealed most clinicians and patients shared opinions or concerns and frequently arrived at an agreed-upon decision, but most observed decisions still fell short of the criteria that constitute SDM (16). In a recent qualitative study among patients with schizophrenia spectrum disorders, including schizoaffective, schizophreniform, schizotypal personality, and delusional disorder, participants reported that healthcare professionals inconsistently involved them in treatment decisions (17). Meanwhile, in a qualitative study of patients' experiences with antipsychotic drugs in Norway, only one-third of the participants reported receiving sufficient information, while the rest received little to no information (49).

The degree to which SDM is relevant and sensitive to the Asian culture and practice is still not well-known. SDM is expected to be less common in Asian culture than in the Western system, which supports individualism, empowerment, and independence (50, 51), whereas health providers in Asian countries are assumed to be more paternalistic in their treatment approaches (52). In Asian clinical settings, mental health professionals are expected to be respected as authority figures, which might make it more difficult for patients to express preferences and discuss treatment options (53). A recent study among people with schizophrenia in China using a qualitative interview explored participants' attitudes, experiences, and factors related to SDM (19). All the participants described situations in which the psychiatrist made the decision, and the family gave informed consent to decision-making. Some participants felt that the psychiatrist dominated the decision-making process without discussing preferences for treatment. Participants felt excluded and that they had no influence over decision-making when the psychiatrist and the family made a joint decision without them. A very recent review recommended family-centered decision-making (FCDM) as a more adaptive approach for use among Asian service users than the usual SDM. FCDM may be seen as allocating a greater degree of priority to patients challenged by more disabling illnesses, such as among patients with schizophrenia (54).

The level of SDM in our study of an Asian population is still comparable to, and relatively higher than, those findings in Western countries, despite the expectation that SDM experiences would be fewer. This can be explained by a few potential reasons. Firstly, about 20% of the participants were excluded from this study for various reasons, such as language barriers and their refusal to participate for unknown reasons. It is possible that among these excluded participants are people who possibly could not comprehend the questionnaires and had a lower capacity for SDM. The results could have been lower if they had been included. Secondly, this hospital caters to a population that is socioeconomically more privileged compared to the general population. The economic backgrounds of the outpatient attendees might be different, since this teaching hospital is semiprivate, unlike government hospitals run by the Ministry of Health where medical services are charged at a minimal rate. Additionally, this hospital is located in an urban area that may cater to people with a higher capacity for SDM. The urban population preferred SDM much more than the rural population, according to one local study (13). Urban dwellers are often younger, more literate, and more highly educated (45). Of our participants, 34% had at least a tertiary education level compared to one local study in a less urbanized population that showed a much smaller tertiary education percentage (6%) (55). Our participants' education level is also higher when it is compared to those in a population-based study using nationwide registers, which occurred in one European country and in which only 12% had a tertiary educational level (56). Our patients' educational backgrounds are similar to those investigated in a study done at the same center on common medical illnesses, which revealed a slightly higher tertiary education level percentage (36%) (57). Patients with higher levels of education and income were shown to prefer autonomous roles in a previous study of the general public (28). Thus, these factors may affect the findings for both the level of SDM and role preference in this current study.

#### Role Preference

Slightly more than half (56%) of the participants in the current study preferred autonomous roles. Role preference measures individual preference in decision-making in terms of whether they prefer autonomous or passive roles. In a systematic review paper, there were emerging trends and perspectives that SDM is generally highly accepted and desirable in the treatment of patients with schizophrenia and related disorders (58). People with schizophrenia were shown to prefer SDM with varying degrees of role preferences, based on a recent qualitative study in China (19). An earlier study in Spain of people with bipolar disorder and schizophrenia revealed a lower percentage (36%) with autonomous role preference (23). Non-psychiatric patients treated in primary care settings were shown to have a much higher autonomous role preference compared to patients with bipolar disorder and schizophrenia, according to a previous study. Non-psychiatric primary care patients were 18 times more likely to prefer to be given options about their treatment and twice as likely to prefer making medical decisions on their own (59). This contrasts with a previous study among inpatients with schizophrenia using Autonomy Preference Index scores, in which patients with schizophrenia had slightly higher mean scores than those reported for the primary care patients (60). Role preference among mental health service users, in general, was reported to be high (82%) in a very recent study whereby the majority of them preferred active and shared decision-making regarding their medication (26). Generally, patients with psychiatric illnesses appeared to prefer autonomous roles.

### Correlation Between Role Preference and Shared Decision-Making Experiences

Another finding from the current study worth discussing is that role preference did not correlate with SDM experiences. Autonomous role preference was considered to be correlated with SDM when the participants who preferred autonomous roles also perceived high SDM experiences. In our study, the majority (60%) of participants who preferred autonomous roles perceived a lack of SDM experiences. Among the total respondents, only 22% of participants matched their autonomous role preference with high SDM experiences. The Hungarian national survey revealed that the preferred and perceived roles matched for 52% of the population, whereas 32% preferred more participation and 16% opted for less (46). Another study revealed a mean of congruence between the preference for and perceived participation in decision-making of 60% (28). However, both studies were conducted on the general population in medical decision-making. A study reviewing major psychiatric illnesses showed that SDM occurs less often in mental health treatment than is desired by patients (58). Another study involving patients with schizophrenia spectrum disorders revealed that almost all participants identified a desire for SDM but nearly all also described experiences in which they felt insufficiently included in treatment-related decisions (17). People diagnosed with schizophrenia perceived they were not involved in the SDM although they may have had a preference for SDM, according to a recent study in China (19).

Barriers to this SDM practice being followed against patients' role preferences should be investigated, and intervention should occur. One recent review summarized that the barriers to SDM for psychiatric medication management were due to patients' lack of confidence and awareness of their rights, limited access to information, poor communication by all parties or either party, and misperceptions about patients' decision-making abilities (61). The most commonly identified barriers were the assumption of hierarchical doctor–patient relationships and the paternalistic views of decision-making in the culture. Particularly among patients with schizophrenia, there is a high societal expectation that psychiatrists should hold statutory powers in the treatment of the condition (47). Barriers to implementing SDM also varied based on place of origin; physicians in the United States mentioned limited time, physicians in Jordan reported that a lack of patient education limits SDM practices, and physicians in Israel reported a lack of communication training (62). Meanwhile, in Malaysia, the barriers were noted to be limited teaching of SDM in undergraduate and postgraduate curricula and a lack of accurate and accessible health information for patients (12). The importance of this study is to understand both the role preference and SDM experiences particularly among patients with schizophrenia. Interestingly, there have not been many quantitative studies on the topic in this population. Understanding the level of SDM and role preference will contribute to guiding future research and the development of clinical practice for this population.

### Associated Factors of Shared Decision-Making

#### Association Between Being Younger and Higher Levels of Shared Decision-Making

Our study revealed that being younger was significantly associated with better SDM experiences after being adjusted for other factors. This finding was similar to a study by De las Cuevas et al. that showed an association between being younger and having better SDM experiences (20). Other studies looked into the association between age and role preference, but without including SDM experience in their studies. In these studies, younger patients were shown to prefer autonomous roles compared to older patients (23, 55). Interestingly, our study revealed the association between age and SDM experiences but not role preference. This might mean that clinicians are giving more opportunities to younger patients to get involved in decision-making regardless of their role preference. The brain changes that happen among patients with schizophrenia are another possible explanation of why the older age-group does not experience SDM as much. White and gray matter deteriorations have been observed in the brains of patients with schizophrenia during late adulthood, with a vulnerability in the prefrontal and cingulate cortices (63). This assumption may prevent clinicians from practicing better SDM with their older patients. However, aging can affect executive functioning differently (64). Thus, clinicians should not underestimate the capacity for SDM among their older patients.

#### Association Between Being a Clozapine User and Lower Shared Decision-Making

Our study also revealed a significant association between being a clozapine user and SDM. The non-clozapine user group had a strong positive correlation with SDM experiences, even after adjustments were made for other factors, as the p = 0.000. Twenty-six percent of our participants were clozapine users, a percentage almost similar to earlier local studies in Malaysia that revealed clozapine user frequency to be 20% (55, 65). All the patients in our study who were prescribed clozapine were being treated as treatment-resistant schizophrenia (TRS) patients, consistent with most guidelines that support the use of clozapine in the management of TRS (66). This association between clozapine users having lower SDM is most probably TRS-related rather than being due to the effect of clozapine. Clozapine has generally been proven to improve cognitive functions and, presumably, the capacity for SDM (67).

TRS reflects a more severe stage of the illness and is associated with more negative symptoms, a longer duration of illness, frequent relapses and hospitalization, more social or occupational dysfunction, a lack of family support, and poor therapeutic alliance (68). All these factors may affect the patients' capacity for SDM. A lack of social support for TRS patients may reduce such an individual's capacity to live more independently in the community environment and have meaningful relationships (69). Poor cognitive impairment is reflective of poor cognitive reserve, which may affect an individual's capacity for interpersonal functioning (70), which happens more so in patients with TRS (71). Self-esteem is another factor that may affect the capacity for SDM with patients with schizophrenia, especially among those with TRS; it impairs psychological well-being and the capacity to express a preference for SDM (72). Therefore, it may be understandable that the TRS patient group has a lower capacity for SDM.

### Associated Factors of Autonomous Role Preference

#### Association Between Lower Levels of Insight and Autonomous Role Preference

Our study revealed a negative correlation between levels of insight and patient role preferences. Patients with lower levels of insight, surprisingly, chose autonomous role preference more than those with better insight. A similar negative correlation was observed between levels of insight and SDM experiences in the simple regression model, but this correlation became insignificant in the multiple regression model when adjusted with other confounders. This finding is similar to that of an earlier study that used a seven-item questionnaire to measure insight and the autonomy preference index to measure role preference (60). However, in this earlier study, the negative correlation between insight and role preference was not significant, with a *p* = 0.09. Other previous studies seem to prove the contrary and show that a lack of insight had the strongest link to a lack of decision-making capacity relating to treatment (29). Poor insight is seen as the most common and absolute barrier to SDM among patients with schizophrenia (32, 47) and has been linked to a poorer perceived therapeutic alliance (73). This can be explained by the description of insight as the ability of people with schizophrenia to recognize that they have an illness and their ability to understand how their experiences relate to the illness (74).

One possible explanation for our finding, which differs from most previous studies, as they were conducted in western countries, is related to Asian cultural values. The paternalistic approach is still very much being practiced and generally accepted by patients and the public. As the patients gain insight, they will fall back on these Asian values in leaving decision-making to the doctors, a process that is socially desirable (60). However, this value is disrupted when their capacity for insight is impaired, and their preference for an autonomous role during this stage may reflect an act of distrust when they are still under the influence of the symptoms of the illness (32). Previous studies looking into the association between the domain-specific insight of patients with schizophrenia and symptomatology, multiple neurocognitive functions, and personality-related traits found that poor insight was shown to be associated with self-certainty, increased novelty-seeking behavior, better self-esteem and self-efficacy, higher education (75), and overconfidence (76). These factors can predispose such people to be more active in SDM despite having poor insight. Thus, they preferred to be more active in decision-making with their clinician.

Nevertheless, helping patients gain insight into their illness is an important process. Insight has been proven to enhance medication adherence and long-term clinical outcomes and offer a better quality of life (77). A recent review in a journal of ethics suggests that a patient's lack of insight should not be a reason for healthcare providers to abandon decision-sharing with a patient (78). It would be ethical for clinicians to improve their patients' insight about the proven benefits and assist autonomous role preference and SDM at the same time in order to facilitate recovery.

#### Association Between Being on Oral Antipsychotics Only and Autonomous Role Preference

In this current study, those on oral antipsychotics only were more likely to have an autonomous role preference compared to those on LAI antipsychotics. In this study setting, patients who are on oral antipsychotics only (65%) may have been in a situation where good responses had already been achieved with oral medication or they had not been keen on LAI for various reasons. One common reason for patients' reluctance to be treated with LAI is the stigma associated with it. A study revealed that patients tend to prefer the route of administration that is commonly used, and that LAIs generate greater feelings of shame or stigma (79). However, the reasons for using the different formulations of medication among the patients were not explored in this study.

The association found in this study between being on oral antipsychotics only and autonomous role preference may be explained by the fact that, in this study setting, LAI is still not commonly used in the early stages of illness. Being on oral antipsychotics only reflects a lower illness severity. It is acknowledged that SGA LAIs are increasingly chosen for use in the early stages of illness due to the advantages they offer in preserving white matter brain volume, which provide a greater degree of neuroprotection and better cognitive performance. A very recent article from Hong Kong provided consensus statements promoting the use of SGA LAIs with all patients with schizophrenia as an SDM process due to the extensive volume of evidence supporting the benefits for treatment outcomes regardless of the illness stage (80). The practice of using SGA LAIs as a preferred option in the early stages of illness is new in Malaysia. Generally, LAIs are still reserved for patients having difficulties in controlling the symptoms of illness. One recent review revealed that patients admit to preferring a more directive/paternalistic practitioner style during a crisis, but they feel pressured or persuaded into accepting pharmacological treatments like LAIs if they fail to take their prescribed oral medication (81). These factors, which can signify more chronic and cognitive impairment, may affect their role preference and subsequently the SDM process. This may explain our findings.

### Strengths, Limitations, and Recommendations

Our study findings confirm previous reports, albeit not many in number, on the lack of SDM practices, despite it being a widely accepted standard of patient-centered care and promoted by the authoritative guidelines. The implementation of SDM among patients with schizophrenia has remained relatively less successful despite the increasing development of SDM interventions (48). This study addresses this gap and highlights some important complexities.

The limitations of our study were related to bias, including selection and response biases. Additionally, this study, being cross-sectional in design, could not establish cause-and-effect relationships between variables. The relatively small sample size of this study also limited the reliability of the study. Clinician perspectives, which may have complemented the findings, were not assessed in this study, as it was limited to the patients' perspectives. The study was conducted in only one center, i.e., a teaching hospital, which may limit the generalizability of the findings. In addition, there were limited factors contributing to SDM that be analyzed in this current study.

Other factors contributing to SDM should be explored in future studies. More research is needed regarding how SDM can be implemented in regular mental health care. A randomized controlled trial with complementary SDM interventions is recommended to yield the maximum effect on patients as active participants (82). Adapting SDM concepts and tools to public mental healthcare settings poses numerous challenges, as reported from the field tests of one of the patient decision aids (PDAs) for consumers, which considered the use of antipsychotic medication (83). Newer PDA tools for aiding antipsychotic medication decision-making were developed by a research team in a study by Zisman-Ilani et. al. (84). This tool was used with patients and by clinicians as part of the psychiatric consultation and was shown to be valuable and acceptable for people with first-episode and long-term psychosis.

Due to cultural differences, locally validated tools should be available. In Malaysia, there has been an ongoing initiative to improve SDM for patients with MDD but none yet directed toward patients with schizophrenia. At the moment of writing, one local trial had just been completed on the strategic alliance between patients and healthcare professionals in recovery (SAPHIR). The intervention groups were given a booklet of scripts for doctors and the Antidepressant PDA Booklet to facilitate SDM during patient–physician consultations. A similar initiative may be applicable for patients with schizophrenia. A recent open forum suggested a new conceptualization, shared risk-taking, to facilitate the implementation of SDM (30). The clinician and patient should explicitly conduct a risk assessment of a decision, its safety implications, and the patient's capacity to be involved in the decision-making process. Most decision support tools, however, are not designed to capture risk-taking in the context of complex decisions with broader life implications.

## Conclusion

The present study showed that the practice of SDM is still lacking in the treatment of patients with schizophrenia in Malaysia, even though many of them preferred to be involved in the decision-making pertaining to their treatment. Contrary to the understanding that the paternalistic approach of decision-making being socially desirable in Asian cultural values, this study illustrated that active involvement in decision-making is preferred by many patients with schizophrenia. This study indicates the need for clinicians to improve the way they involve patients in their treatment process. More research is needed regarding how SDM can be implemented with patients with schizophrenia, especially in Asian population settings. Additionally, the chronicity among patients with schizophrenia, as reflected by being in the TRS group and older in age, may contribute to a lack of SDM. The coincidental finding connecting a lower insight level and being on oral antipsychotics only with more autonomous role preference warrants further study and a better explanation.

## Data Availability Statement

The raw data supporting the conclusions of this article will be made available by the authors, without undue reservation.

## Ethics Statement

This study was approved by the Universiti Kebangsaan Malaysia (UKM) research ethics committee (JEP-2019-530). The participants provided their written informed consent to participate in this study.

## Author Contributions

MI contributed to the original design of the study, management of the study, data collection, data analysis, interpretation of results, preparation, and editing of the manuscript. MM contributed to the conceptualization of the study, interpretation of results, and review of the manuscript. Both authors read and approved the final manuscript.

## Conflict of Interest

The authors declare that the research was conducted in the absence of any commercial or financial relationships that could be construed as a potential conflict of interest.

## Publisher's Note

All claims expressed in this article are solely those of the authors and do not necessarily represent those of their affiliated organizations, or those of the publisher, the editors and the reviewers. Any product that may be evaluated in this article, or claim that may be made by its manufacturer, is not guaranteed or endorsed by the publisher.
